# Magnetic resonance parametric mapping of the spleen for non-invasive assessment of portal hypertension

**DOI:** 10.1007/s00330-020-07080-5

**Published:** 2020-08-04

**Authors:** Narine Mesropyan, Alexander Isaak, Anton Faron, Michael Praktiknjo, Christian Jansen, Daniel Kuetting, Carsten Meyer, Claus C. Pieper, Alois M. Sprinkart, Johannes Chang, Burkhard Maedler, Daniel Thomas, Patrick Kupczyk, Ulrike Attenberger, Julian A. Luetkens

**Affiliations:** 1 Department of Diagnostic and Interventional Radiology and Quantitative Imaging Lab Bonn (QILaB), University Hospital Bonn, Venusberg-Campus 1, 53127 Bonn, Germany; 2 Department of Internal Medicine I, University Hospital Bonn, Venusberg-Campus 1, 53127 Bonn, Germany; 3 Philips GmbH Germany, Roentgenstrasse 22, 22335 Hamburg, Germany

**Keywords:** Liver cirrhosis, Portal, Hypertension, Magnetic resonance imaging

## Abstract

**Objectives:**

In patients with advanced liver disease, portal hypertension is an important risk factor, leading to complications such as esophageal variceal bleeding, ascites, and hepatic encephalopathy. This study aimed to determine the diagnostic value of T1 and T2 mapping and extracellular volume fraction (ECV) for the non-invasive assessment of portal hypertension.

**Methods:**

In this prospective study, 50 participants (33 patients with indication for trans-jugular intrahepatic portosystemic shunt (TIPS) and 17 healthy volunteers) underwent MRI. The derivation and validation cohorts included 40 and 10 participants, respectively. T1 and T2 relaxation times and ECV of the liver and the spleen were assessed using quantitative mapping techniques. Direct hepatic venous pressure gradient (HVPG) and portal pressure measurements were performed during TIPS procedure. ROC analysis was performed to compare diagnostic performance.

**Results:**

Splenic ECV correlated with portal pressure (*r* = 0.72; *p* < 0.001) and direct HVPG (*r* = 0.50; *p* = 0.003). No significant correlations were found between native splenic T1 and T2 relaxation times with portal pressure measurements (*p* > 0.05, respectively). In the derivation cohort, splenic ECV revealed a perfect diagnostic performance with an AUC of 1.000 for the identification of clinically significant portal hypertension (direct HVPG ≥ 10 mmHg) and outperformed other parameters: hepatic T2 (AUC, 0.731), splenic T2 (AUC, 0.736), and splenic native T1 (AUC, 0.806) (*p* < 0.05, respectively). The diagnostic performance of mapping parameters was comparable in the validation cohort.

**Conclusion:**

Splenic ECV was associated with portal pressure measurements in patients with advanced liver disease. Future studies should explore the diagnostic value of parametric mapping accross a broader range of pressure values.

**Key Points:**

*• Non-invasive assessment and monitoring of portal hypertension is an area of unmet interest.*

*• Splenic extracellular volume fraction is strongly associated with portal pressure in patients with end-stage liver disease.*

• *Quantitative splenic and hepatic MRI-derived parameters have a potential to become a new non-invasive diagnostic parameter to assess and monitor portal pressure.*

## Introduction

Any chronic liver disease may lead to liver fibrosis, which distorts normal liver architecture by the expansion of the extracellular space, and impairs hepatic function [1]. Liver cirrhosis is tightly linked to the occurrence of portal hypertension [2]. Portal hypertension may lead to life-threatening complications such as esophageal variceal bleeding, ascites, and hepatic encephalopathy. Therapy refractory ascites is associated with significantly increased mortality 6–12 months after diagnosis [3]. Therefore, precise diagnosis of portal hypertension plays an important role in clinical decision-making and early interventions may prevent severe complications. Currently, the hepatic venous pressure gradient (HVPG) is considered the reference standard for the assessment of portal hypertension [4]. The HVPG is the difference between the wedged portal vein pressure and the free hepatic venous pressure. Portal hypertension is defined as HVPG > 5 mmHg. Clinically significant portal hypertension is defined as an increase in HVPG to ≥ 10 mmHg [5, 6]. The invasive procedure of HVPG has clear disadvantages because it may be associated with procedural complications and, therefore, cannot be used as a follow-up method. HVPG measurements also require high clinical expertise and are costly. Therefore, alternative non-invasive techniques are needed for the assessment and monitoring of portal pressure.

Recently, quantitative T1 and T2 mapping techniques have been applied to the liver and spleen and might be used for tissue characterization and for the staging of liver fibrosis [7, 8]. Furthermore, T1 relaxation times can also be measured before and after the administration of an extracellular contrast agent, which allows the additional calculation of the extracellular volume (ECV). ECV values are calculated from the change in relaxation rate (R1 = 1/T1) of blood and parenchyma corrected for the hematocrit [9]. ECV is a measure of the extracellular space and represents the tissue volume, which is not taken by cells [10]. Also, ECV is a physiologically intuitive unit of measurement and is independent of field strength. ECV was initially developed for quantifying the myocardial extracellular fractional distribution volume and has been validated in histopathological studies as a measurement of diffuse myocardial fibrosis [11]. Besides the evaluation of myocardial tissue composition, this technique can also be used as a new tool for the non-invasive assessment of liver fibrosis [7, 8]. Furthermore, animal studies suggest that abdominal ECV measures might be correlated with portal pressure measurements [8]. Also, splenic post contrast T1 measurements showed correlations with HVPG in humans [12]. Therefore, the assessment of splenic ECV might be advantageous for non-invasive assessment of portal pressure, as splenomegaly in portal hypertension is not only caused by congestion but also by tissue hyperplasia and fibrosis [13, 14]. The purpose of our study was to find a possible correlation between different parametric magnetic resonance imaging (MRI) parameters (T1, T2, and ECV mapping) of liver and spleen and to evaluate their diagnostic performance for the assessment of portal hypertension.

## Material and methods

This prospective, proof-of-concept study was approved by the institutional review committee. All study participants provided written informed consent prior to MRI examination. From November 2018 to September 2019, patients with advanced liver disease and portal hypertension scheduled for trans-jugular intrahepatic portosystemic shunt (TIPS) implantation were consecutively included in this study. All patients completed MRI before TIPS implantation. Healthy volunteers underwent MRI as controls. Diagnosis of refractory ascites was based on the diagnostic criteria recommended by the International Ascites Club (IAC) [15]. Clinical data and laboratory markers were retrieved from the institutional medical information system. The control group consisted of healthy volunteers with no previous medical history of liver disease. All control participants had normal liver MRI and normal laboratory results and were defined to have normal portal pressure.

### Magnetic resonance imaging

All imaging was performed on a clinical whole-body 1.5-T MRI system (Ingenia, Philips Healthcare) equipped with 32-channel abdominal coil with digital interface for signal reception. Besides morphological sequences, patients underwent parametric mapping MRI of the liver and the spleen: For splenic and hepatic T1 mapping, a heart rate independent10-(2)-7-(2)-5-(2)-3-(2) modified Look-Locker inversion recovery (MOLLI) acquisition scheme [16] with internal triggering was implemented. The following technical parameters were applied: time of repetition (TR) 1.92 ms, time of echo (TE) 0.84 ms, flip angle (FA) 20°, parallel imaging factor 2, acquired voxel size 1.98 × 2.45 × 10 mm, reconstructed voxel size 1.13 × 1.13 × 10 mm, scan duration/breath-hold 14.0 s. Using the same technique, post-contrast T1 maps were performed in the same positions as pre-contrast examinations. As ECV measurements in the liver are constant from 5 to 25 min according to the experimental data, post-contrast T1 mapping was performed 10 min after contrast administration [17]. For contrast enhancement, the extracellular contrast agent Gadobutrol (0.2 mmol per kilogram of body weight, Gadovist, Bayer Healthcare Pharmaceuticals) was injected at a rate of 1.5 ml/s. T2 mapping was performed before contrast administration using a six-echo gradient spin echo sequence (GraSE) [18]. The following scan parameters were applied: TR 450 ms, inter-echo spacing 16 ms, FA 90°, parallel imaging factor 2.5, acquired voxel size 1.98 × 2.01 × 10  mm, reconstructed voxel size 0.88 × 0.88 × 10 mm, scan duration/breath-hold 15/3 × 5 s. Parametric maps were acquired in a single transverse section at the level of the bifurcation of portal vein covering the liver and the spleen. T1 and T2 relaxation maps were reconstructed at the scanner console.

### HVPG measurements by trans-jugular intrahepatic portosystemic shunt implantation

TIPS procedures establish an artificial connection between the portal and systemic circulation. It is applied in patients with end-stage liver disease for reduction of the portal pressure [15]. TIPS procedure was performed in aseptic conditions under fluoroscopic guidance by experienced interventional radiologists. After puncture of the right portal vein branch, a guidewire was advanced through the TIPS needle and advanced into the portal vein. Afterward, an angiographic 5-French pigtail was advanced into the portal vein for direct portal pressure measurement. No wedged portal vein pressure was measured in this study. Central vein pressure was in inferior vena cava. Direct HVPG was calculated as a difference between portal vein pressure and free inferior vena cava pressure. Also, absolute portal vein pressure was recorded. Significant portal hypertension was defined as a direct HVPG of ≥ 10 mmHg. No invasive portal pressure measurements were performed in the control group. Healthy controls were defined to have no portal hypertension.

### Image analysis

Image analyses were performed by an experienced board-certified radiologist, blinded to the clinical information and portal vein pressure measurements. Three regions of interest (ROIs) were respectively drawn within the liver and the spleen parenchyma, away from confounding factors like vessels, biliary structures, and organ boundaries. Minimum ROI size was ≥ 1 cm^2^. The ROIs were firstly placed into the native T1 map. Afterward, the ROIs were copied on all other relaxation maps for the same patient. Mean T1 and T2 relaxation times were used for analysis. T1 values of the blood pool were obtained from the abdominal aorta. ECV values were normalized for hematocrit and calculated from pre- and post-contrast T1 values using the following equation [9]: ECV = (1 − hematocrit)*(1/T1 parenchyma post-contrast −1/T1 parenchyma pre-contrast)/(1/T1 aortic post-contrast −1/T1 aortic pre-contrast). For this explorative study, we assumed that for abdominal ECV calculations, a bolus-only contrast injection technique leads to a dynamic equilibrium 10 min after contrast administration [17]. Blood hematocrit levels were obtained before MRI investigations.

### Statistical analysis

Statistical analysis was performed using SPSS Statistics (Version 22, IBM) and MedCalc (version 19.1.3, MedCalc Software). Patient characteristics are presented as mean ± standard deviation or as absolute frequency. Continuous variables between the two groups were compared by using the Student *t* test. Dichotomous variables were compared by using the *χ*2 test. Pearson correlation coefficient (*r*) was used for correlation analyses. In the derivation cohort, the diagnostic performance of MRI parameters was analyzed by plotting receiver operating characteristics and comparing the area under the curve (AUC). Youden’s index was used to determine the optimal cutoff of the ROC curve providing the highest combination of sensitivity and specificity. The presence of clinically significant portal hypertension (direct HVPG ≥ 10 mmHg) was the reference standard against which the diagnostic performance of MRI-derived mapping parameters of spleen and liver was tested. AUCs were compared by using the method proposed by DeLong et al [19]. Using the cutoff values of the derivation cohort, sensitivity, specificity, accuracy, and predictive values were calculated for the validation cohort. The level of statistical significance was set to *p* < 0.05.

## Results

### Cohort characteristics

A total of 33 patients with liver cirrhosis and refractory ascites/esophageal variceal bleeding and 17 healthy volunteers were included. The first 40 participants (28 patients with liver cirrhosis and 12 healthy volunteers) were used as a derivation cohort to establish the cutoff values of mapping parameters. The next 10 participants that were included constituted our validation cohort (5 patients with liver cirrhosis and 5 healthy volunteers). The mean interval between pre-interventional MRI and TIPS implantation was 9.66 ± 11.87 days. All patients had clinically significant portal hypertension (direct HVPG ≥ 10 mmHg). There were no peri- or post-procedural complications related to TIPS implantation.

#### Derivation cohort

Etiologies of liver disease included alcoholic liver disease (*n* = 14, 50.00%), non-alcoholic fatty liver disease (NAFLD, *n* = 2, 7.14%), virus-related liver cirrhosis (*n* = 1, 3.57%), toxic liver disease (*n* = 3, 10.71%), unknown etiology (*n* = 5, 17.85%), and sinusoidal liver disease (*n* = 2, 7.14%). Indications for TIPS implantation were refractory ascites (*n* = 21/28, 75.00%) and esophageal variceal bleeding (*n* = 7/28, 25.00%).

#### Validation cohort

Etiologies of liver disease included alcoholic liver disease (*n* = 4, 80.00%) and unknown etiology (*n* = 1, 20.00%). Indications for TIPS implantation were refractory ascites (*n* = 4/5, 80.00%) and esophageal variceal bleeding (*n* = 1/5, 20.00%). The clinical characteristics of the derivation and validation cohorts are summarized in Table 1.

**Table 1:** Clinical characteristics of the derivation and validation cohort for patients with clinically significant portal hypertension and healthy control participants

	Derivation cohort (*n* = 40)			Validation cohort (*n =* 10)		
Variable	Healthy controls (*n* = 12)	Portal hypertension (*n* = 28)	*p* value	Healthy controls (*n* = 5)	Portal hypertension (*n* = 5)	*p* value
Age (years)	43.58 ± 17.42	58.32 ± 11.66	0.017	52.40 ± 20.26	55.60 ± 4.50	0.739
Body mass index (kg/m^2^)	22.53 ± 4.57	24.58 ± 5.42	0.281	22.72 ± 2.22	25.88 ± 8.71	0.454
Sex			0.722			0.350
Male	8 (66.66%)	21 (75.00%)		1 (20.00%)	3 (60.00%)	
Female	4 (33.33%)	7 (25.00%)		4 (80.00%)	2 (40.00%)	
Hematocrit level (%)	45.25 ± 3.71	29.46 ± 5.90	< 0.001	41.00 ± 2.55	33.00 ± 3.31	0.003
MELD	6.8 ± 1.4	12.92 ± 5.46	< 0.001	6.20 ± 0.44	13.50 ± 5.00	0.013
CHILD			< 0.001			0.007
A	0 (0.00%)	7 (25.00%)		0 (0.00%)	2 (40.00%)	
B	0 (0.00%)	17 (60.71%)		0 (0.00%)	3 (60.00%)	
C	0 (0.00%)	4 (14.28%)		0 (0.00%)	0 (0.00%)	
Bilirubin (mg/dl)	0.69 ± 0.26	0.137 ± 1.36	0.017	0.39 ± 0.18	1.25 ± 0.71	0.031
ALT (U/l)	35.00 ± 12.67	25.93 ± 9.90	0.041	35.60 ± 30.22	39.40 ± 24.70	0.833
AST (U/l)	23.66 ± 4.57	47.14 ± 27.19	< 0.001	26.00 ± 12.58	61.20 ± 27.39	0.031
GGT (U/l)	36.16 ± 20.78	140.53 ± 240.42	0.031	69.20 ± 94.05	235.20 ± 235.62	0.182
Platelets cells×10^9^/l	281.25 ± 85.24	153.04 ± 112.11	0.001	287.40 ± 39.91	172.50 ± 90.98	0.037
C-reactive protein level (mg/l)	1.09 ± 1.94	10.65 ± 11.82	< 0.001	1.12 ± 0.68	15.68 ± 4.90	0.003
AP (U/l)	49.20 ± 20.42	121.64 ± 102.18	0,035	83.60 ± 27.71	139.40 ± 60.85	0.099
Creatinine (mg/dl)	0.88 ± 0.21	1.14 ± 0.54	0.042	0.78 ± 0.13	2.25 ± 2.18	0.170
Albumin (g/l)	Not available	31.90	–	Not available	32.98 ± 6.91	–
International normalized ratio	1.01 ± 0.04	1.15 ± 0.09	< 0.001	1.26 ± 0.58	1.30 ± 0.18	0.887

Continuous data are means ± standard deviations. Nominal data are absolute frequencies with percentages in parentheses. *Abbreviations*: *MELD*, score model of end-stage liver disease; *CHILD*, Child-Pugh score; *ALT*, alanine aminotransferase; *AST*, aspartate aminotransferase; *AP*, alkaline phosphatase; *GGT*, gamma-glutamyltransferase

### MRI results

**Fig. 1:**
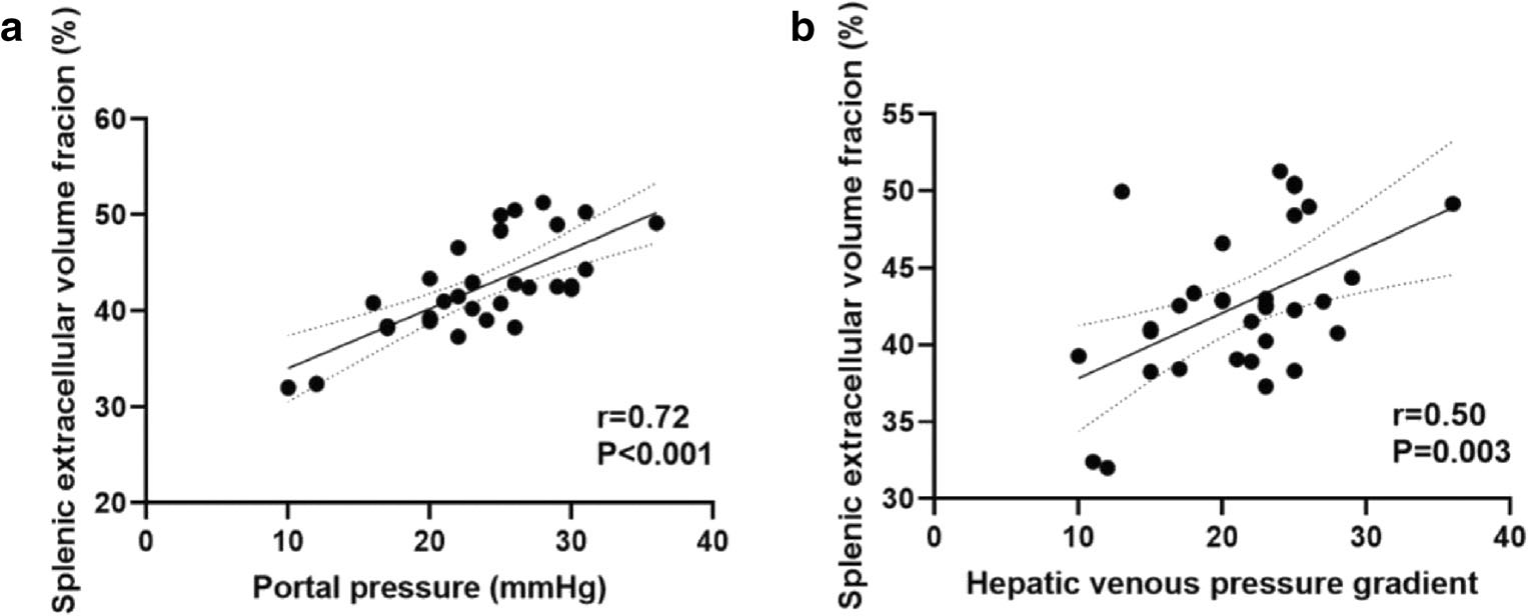
Scatter plots shows correlations between splenic extracellular volume fraction and portal vein pressure (**a**) and direct hepatic venous pressure gradient (**b**) in the patients with liver cirrhosis (*n* = 33). Regression line is given with 95% confidence interval

Portal vein pressure (*r* = 0.72, *p* < 0.001) and direct HVPG (*r* = 0.50, *p* = 0.003) were significantly correlated with splenic ECV in cirrhotic patients (see Fig. 1). A correlation matrix is given in Table 2.

#### Derivation cohort

Compared with healthy controls, patients with liver cirrhosis had significant increased splenic native T1 relaxation times (1010.17 ± 49.13 ms vs. 1100.52 ± 95.76 ms; *p* < 0.001), T2 relaxation times (98.83 ± 11.69 ms vs. 113.17 ± 18.72 ms, *p* = 0.007), and splenic ECV values (25.82 ± 2.40% vs. 42.53 ± 6.29%; *p* < 0.001). There were significant differences in hepatic MRI parameters between controls and patients: native T1 relaxation time (544.78 ± 41.25 ms vs. 681.03 ± 83.93 ms; *p* < 0.001) and ECV (26.14 ± 2.31% vs. 45 ± 18.55%; *p* < 0.001). No significant differences in hepatic T2 relaxation times were present between both groups (48.58 ± 8.41 ms vs. 53.72 ± 7.56 ms; *p* = 0.062).

#### Validation cohort

Splenic and hepatic MRI results of the validation cohort are given in Table 3.

**Table 2:** Correlation matrix for quantitative MRI parameters and parameters of portal pressure of the patients with liver cirrhosis

	Splenic native T1		Splenic post-contrast T1		Splenic T2		Splenic ECV		Hepatic native T1		Hepatic T2		Hepatic ECV	
Variable	*r* value	*p* value	*r* value	*p* value	*r* value	*p* value	*r* value	*p* value	*r* value	*p* value	*r* value	*p* value	*r* value	*p* value
Portal vein pressure	0.31	0.091	−0.300	0.101	0.19	0.294	0.72	< 0.001	0.025	0.894	0.02	0.925	0.07	0.714
Direct HVPG	0.28	0.122	−0.076	0.679	0.17	0.361	0.50	0.003	−0.19	0.918	−0.83	0.648	−0.12	0.945

*Abbreviations*: *ECV*, extracellular volume fraction; *HVPG*, hepatic venous pressure gradient

**Table 3:** Splenic and hepatic MRI characteristics of the derivation and validation cohort for patients with portal hypertension and healthy participants

	Derivation cohort (*n* = 40)			Validation cohort (*n* = 10)		
Variable	Healthy controls (*n* = 12)	Portal hypertension (*n* = 28)	*p* value	Healthy controls (*n* = 5)	Portal hypertension (*n* = 5)	*p* value
Hepatic venous pressure gradient	Not available	20.71 ± 5.49	–	Not available	22.40 ± 7.83	–
Portal pressure (mmHg)	Not available	23.07 ± 5.47	–	Not available	26.00 ± 7.17	–
Splenic native T1 relaxation time (ms)	1010.17 ± 49.13	1100.52 ± 95.76	< 0.001	1016 ± 45.05	1116 ± 23.02	0.002
Splenic contrast T1 relaxation time (ms)	360.92 ± 51.41	291.44 ± 56.89	0.001	483.00 ± 76.34	333.00 ± 78.36	0.002
Splenic extracellular volume fraction (%)	25.82 ± 2.40	42.53 ± 6.29	< 0.001	28.94 ± 1.53	42.89 ± 3.92	< 0.001
Splenic T2 relaxation time (ms)	98.83 ± 11.69	113.17 ± 18.72	0.007	103.00 ± 5.19	116 ± 13.21	0.062
Hepatic native T1 relaxation time (ms)	544.78 ± 41.25	681.03 ± 83.93	< 0.001	535.60 ± 20.88	705.60 ± 59.18	< 0.001
Hepatic extracellular volume fraction (%)	26.14 ± 2.31	45.00 ± 18.55	< 0.001	27.35 ± 4.22	35.42 ± 6.99	< 0.001
Hepatic T2 relaxation time (ms)	48.58 ± 8.41	53.72 ± 7.56	0.062	47.60 ± 2.07	54.66 ± 3.51	0.005

Continuous data are means ± standard deviations

**Table 4:** Diagnostic performance of different quantitative MRI parameters of the derivation and validation cohort for assessment of clinically significant portal hypertension in patients with advanced liver disease

Variable	Cutoff value	Sensitivity (%)	Specificity (%)	PPV (%)	NPV (%)	Accuracy (%)
Derivation cohort (*n* = 40)						
Splenic extracellular volume fraction (%)	> 30.42%	100.0 (87.5–100.0)	100 (75.8–100.0)	100.0 (87.5–100.0)	100.0 (75.8–100.0)	100.0 (91.0–100.0)
Splenic native T1 (ms)	> 1060 ms	74.07 (55.3–86.8)	91.67 (64.6–98.5)	95.2 (77.3-99.2)	61.1 (38.6–79.7	79.5 (64.5–89.2)
Splenic T2 (ms)	> 115 ms	46.4 (29.5–64.2)	100 (75.8–100)	100.0 (77.2–100.0)	44.4 (27.6–62.7)	62.5 (47.0–75.8)
Hepatic extracellular volume fraction (%)	> 29%	89.29 (72.8–96.3)	91.67 (64.6–98.5)	96.2 (81.1–99.3)	78.6 (52.4–92.4)	90.0 (76.9–96.0)
Hepatic native T1 (ms)	> 569 ms	92.86 (77.4–98.0)	91.67 (64.6–98.5)	96.3 (81.7–99.3)	84.6 (57.8–95.7)	92.5 (80.1–97.4)
Hepatic T2 (ms)	> 47.60 ms	85.71 (68.5–94.3)	58.33 (32.0–80.7)	82.8 (65.5–92.4)	63.6 (35.4–84.8)	77.5 (62.5–87.7)
Validation cohort (*n* = 10)						
Splenic extracellular volume fraction (%)	> 30.42%	100 (56.6–100.0)	80.00 (37.6–96.4)	83.3 (43.6–97.0)	100 (51.0–100.0)	90.0 (59.6–98.2)
Splenic native T1 (ms)	> 1060 ms	100.0 (56.6–100.0)	80.00 (37.6–96.4)	83.3 (43.6–97.0)	100 (51.0–100.0)	90.0 (59.6–98.2)
Splenic T2 (ms)	> 115 ms	40.0 (11.8–76.9)	100 (56.6–100.0)	100 (34.2–100.0)	62.5 (30.6–86.3)	70.0 (39.7–89.2)
Hepatic extracellular volume fraction (%)	> 29%	80.0 (37.6–96.4)	60.0 (23.1–88.2)	66.7 (30.0–90.3)	75.0 (30.1–95.4)	70.0 (39.7–89.2)
Hepatic native T1 (ms)	> 569 ms	100.0 (56.6–100.0)	100.0 (56.6–100.0)	100.0 (56.6–100.0)	100.0 (56.6–100.0)	100.0 (72.2–100.0)
Hepatic T2 (ms)	> 47.60 ms	100 (56.6–100.0)	40.0 (11.8–76.9)	62.5 (30.6–86.3)	100.0 (34.2–100.0)	70.0 (39.7–89.2)

Cutoff values of the derivation cohort were used to calculate sensitivity, specificity, PPV, NPV, and accuracy of the validation cohort. *Abbreviations*: *PPV*, positive predictive value; *NPV*, negative predictive value. Data in parentheses are 95% confidence interval

### Diagnostic performance of parametric mapping parameters

Several parametric mapping parameters were evaluated regarding the diagnostic performance to diagnose clinically significant portal hypertension.

#### Derivation cohort

**Fig. 2:**
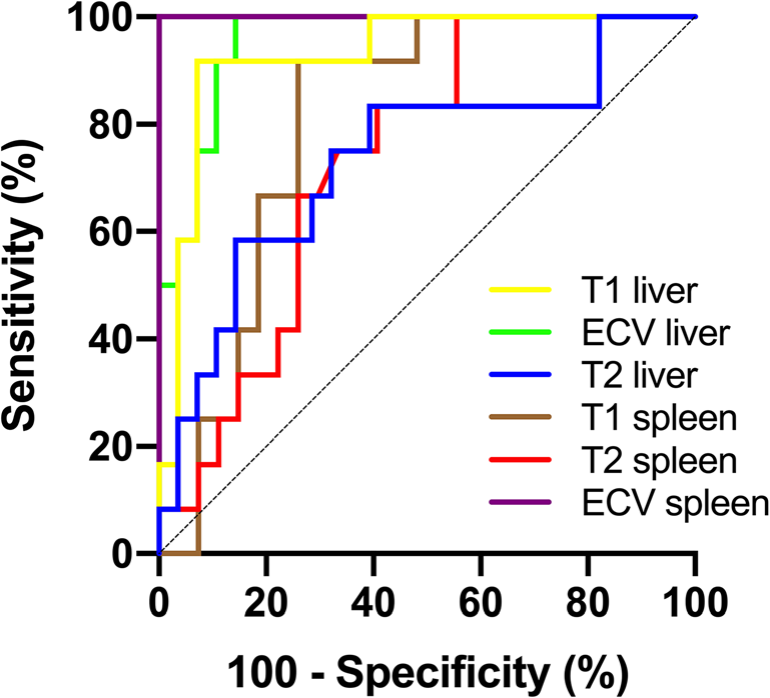
Graphs show receiver operating characteristic curves for diagnosis of clinically significant portal hypertension (direct hepatic venous pressure gradient, ≥ 10 mmHg) in the derivation cohort. Curves are given for hepatic T1 relaxation times (area under curve [AUC], 0.926), hepatic ECV (AUC, 0.954), hepatic T2 relaxation times (AUC, 0.731), splenic T1 relaxation times (AUC, 0.806), splenic T2 relaxation times (AUC, 0.736), and splenic ECV (AUC, 1.000)

**Fig. 3:**
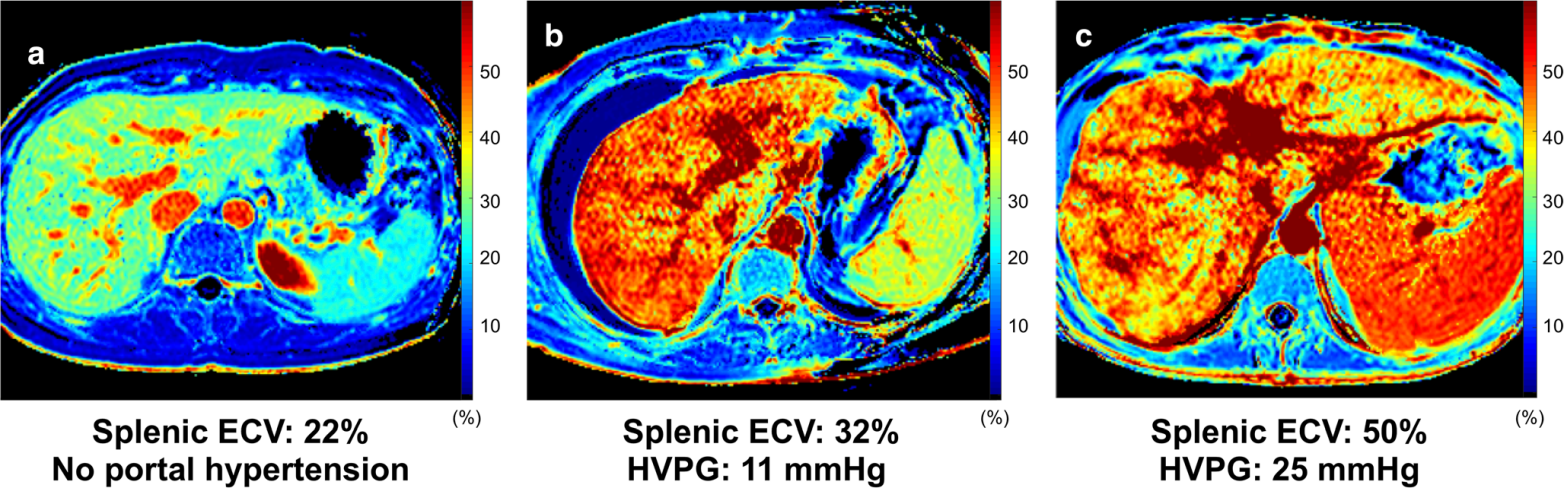
Representative images of splenic extracellular volume (ECV) maps from a healthy volunteer (**a**) and patients with clinically significant portal hypertension (**b**, **c**). *Abbreviations*: ECV, extracellular volume fraction; HVPG, hepatic venous pressure gradient

Splenic ECV revealed a perfect diagnostic performance with an area under the curve (AUC) of 1.000, a sensitivity of 100%, and a specificity of 100% (see Fig. 2, Fig. 3, and Table 4). There were no significant differences in the diagnostic performance of splenic and hepatic ECV (AUC, 1.000 vs. 0.954; *p* = 0.116). The diagnostic performance of splenic ECV was also not significantly higher compared with that of hepatic native T1 (AUC, 1.000 vs. 0.926; *p* = 0.105) but significantly higher than that of splenic native T1 (AUC, 1.000 vs. 0.806; *p* = 0.005). There were no significant differences in the diagnostic performance of native splenic and hepatic T1 (AUC, 0.806 vs. 0.926, *p* = 0.058). Hepatic ECV showed a higher diagnostic performance compared with native splenic T1 (AUC, 0.954 vs. 0.806, *p* = 0.038). Between hepatic ECV and hepatic native T1, no significant differences in diagnostic performance were observed. The diagnostic performance of hepatic and splenic T2 was significantly lower than that of the splenic and hepatic native T1 and ECV parameters.

#### Validation cohort

The parameters of the diagnostic performance of the validation cohort are given in Table 4. The 95% confidence intervals of diagnostic performance were comparable between the derivation and the validation cohort.

## Discussion

In our proof-of-principle study, we evaluated different parametric MRI parameters for non-invasive assessment of portal hypertension. The main findings of our study are that (1) splenic ECV showed a statistically significant correlation with portal pressure and direct HVPG and, (2) splenic and hepatic ECV showed a high diagnostic performance to diagnose clinically significant portal hypertension and performed better than native T1 and T2 mapping parameters.

Hepatocyte injury in chronic liver disease leads can activate potentially fibrogenic cells and promote the occurrence of liver fibrosis. The formation of liver fibrosis leads to an increased deposition of abnormal extracellular matrix components [20]. Liver fibrosis also leads to an increased accumulation of extracellular MRI contrast agents in the extracellular space, which can be measured via ECV assessment. Histopathological studies show correlations between hepatic T1, T2, and ECV with liver fibrosis, as well as hepatic ECV and portal pressure in both animal and human models [8, 13, 14, 21–23]. There are also studies mentioning positive correlations between mapping parameters and magnetic resonance elastography (MRE) [22, 24, 25].

Portal hypertension initially develops because of increased intrahepatic resistance to the passage of blood flow through the liver as a consequence of hepatic fibrotic changes. In this regard, splenomegaly in liver disease is likely to be a consequence of portal congestion with blood pooling as well as pulp hyperplasia and fibrosis [26]. All these changes lead to an expansion of the extracellular space, which is reflected in increased splenic ECV, as shown in our data. In our study, splenic ECV values were also significantly correlated with portal pressure and with direct HVPG.

To our knowledge, there are still no studies directly showing correlations between invasive measured direct HVPG in patients with end-stage liver disease and clinically significant portal hypertension and splenic ECV as a non-invasive marker of portal pressure. Splenic ECV showed a perfect diagnostic performance for clinically significant portal hypertension with an AUC of 1.000 in the derivation cohort. Moreover, although not statistically significant, the diagnostic performance of splenic ECV was superior to that of the liver, possibly because liver fibrosis reflects only one element of pathophysiological changes in portal hypertension, while splenic ECV directly reflects all consequences of portal hypertension (congestion, tissue hyperplasia, and fibrosis). ECV measurements also showed a higher diagnostic performance compared with T1 and T2 mapping parameters, probably because ECV is a physiologically normalized measure and reflects changes of splenic parenchyma more accurately. Therefore, ECV seems to be a stable and biologically significant biomarker for non-invasive assessment of portal hypertension.

Splenic post-contrast T1 has also been recognized as a potential biomarker for diagnosing portal hypertension as well as for treatment monitoring and prognosis [12]. In contrast to the previous study, in which post-contrast splenic T1 values showed a significant correlation with HVPG (*r* = 0.69, *p* = 0.001), we did not found significant correlations between the post-contrast splenic T1 and direct HVPG measures. This might be explained due to our very homogeneous patient collective with end-stage liver disease and clinically significant portal hypertension (mean direct HVPG 20.71 ± 5.49 mmHg). In the previous study, only half of the patients (47%) had clinically significant portal hypertension [12], which might have led to an artificial increase in the correlation coefficient. On the other hand, post-contrast T1 values are known to vary depending on the gadolinium dose, renal clearance rate, scanning time, body composition, and hematocrit levels. The above factors might have contributed to the fact that no significant correlation in post-contrast splenic T1 values was revealed in our study.

Unlike ECV, native hepatic T1 (AUC 0.926) performed better than native splenic T1 (AUC 0.806) in the derivation cohort. This might be explained by a higher contribution of fibrosis to changes in T1 values, as fibrotic changes are more remarkable in hepatic than in splenic parenchyma. Splenic and hepatic T2 mapping parameters had a similar diagnostic performance to patients diagnosed with clinically significant portal hypertension with AUCs of 0.736 and 0.731, respectively. According to previous cardiac studies, increased T2 relaxation times are mainly driven due to myocardial edema or inflammation [27]. Therefore, increased T2 relaxation times in abdominal mapping probably reflect the coexistence of inflammatory or edematous changes in regions of fibrosis [28, 29], which does not correlate well with measures of portal hypertension.

However, hepatic and splenic mapping is a rapidly evolving field and standardized protocols are still being established. Unlike CT, MRI techniques have the advantages that they do not require radiation dose for the assessment of portal hypertension [30]. Also, in contrast to other techniques like MR elastography, which can also be used for the prediction of esophageal varices and, therefore, the severity of portal hypertension [31], the proposed mapping techniques do not require additional equipment. Multiparametric MRI with T1 mapping techniques may reduce the need for invasive and expensive procedures, such as HVPG measurements, in clinical practice. Our findings suggest that MRI-derived ECV values may be a potential new biomarker to assess and monitor portal pressure.

Despite the advantages of ECV measurements as a potential non-invasive parameter, our study has several limitations. First, only patients with end-stage liver disease and significant portal hypertension were included in our explorative study. Therefore, only a small homogenous population with HVPG ≥ 10 mmHg was observed and no conclusion about a broader range of portal pressure measurement can be drawn. As direct portal pressure measurements in patients without indication for TIPS procedure are not clinical routine in our clinic, diagnostic performance of mapping parameters was tested against a control group and not against a patient group without a significant portal hypertension. Therefore, the selected study design does not represent a real-life setting and reported parameters of diagnostic performance have to be regarded as study specific. T1 and T2 maps were acquired in a single transverse section at the level of the bifurcation of portal vein and, therefore, may have missed other significant changes, which probably occurred in other planes. Furthermore, our T1 measurements were not corrected for hepatic steatosis or hepatic/splenic iron overload, which might impair the correct assessment of T1 values [29, 30]. Another limitation of our study was that the reading of all cases was performed only by one experienced radiologist. We used a double-contrast bolus for ECV calculations, as commonly used for cardiac applications. However, as contrast dosage might influence ECV calculation, attention should be paid to standardized contrast protocols, when introducing this technique into clinical routine. The study results have to be considered preliminary and further prospective studies are necessary to establish the results of this study and confirm the accuracy and usefulness of ECV and other MRI parameters for the assessment and follow-up in patients with portal hypertension in routine clinical practice.

In conclusion, in our prospective proof-of-principle study, quantitative splenic and hepatic MRI-derived parameters including ECV appear to be a new valuable, non-invasive diagnostic parameter for the assessment of portal pressure in patients with advanced liver disease. Especially, splenic ECV might provide important information about the presence of clinically significant portal hypertension.

## Abbreviations

BMI: Body mass index
CHILD: Child-Pugh score
ECV: Extracellular volume fraction
GraSE: Gradient spin echo sequence
HVPG: Hepatic venous pressure gradient
IAC: International Ascites Club
MELD: Model for end-stage liver disease
MOLLI: Modified Look-Locker inversion recovery
MRE: Magnetic resonance elastography
NAFLD: Non-alcoholic fatty liver disease
TIPS: Trans-jugular intrahepatic portosystemic shunt
